# 5-(2,4-Dichloro­phen­yl)-3-(4-nitro­phen­yl)-1,2,4-oxadiazole

**DOI:** 10.1107/S1600536810011153

**Published:** 2010-04-28

**Authors:** Hoong-Kun Fun, Mohd Mustaqim Rosli, Sankappa Rai, Arun M Isloor, Prakash Shetty

**Affiliations:** a X-ray Crystallography Unit, School of Physics, Universiti Sains Malaysia, 11800 USM, Penang, Malaysia; b Syngene International Ltd, Biocon Park, Plot No. 2 & 3, Bommasandra 4th Phase, Jigani Link Rd, Bangalore 560 100, India; c Department of Chemistry, Organic Chemistry Division, National Institute of Technology-Karnataka, Surathkal, Mangalore 575 025, India; d Department of Printing, Manipal Institute of Technology, Manipal 576 104, India

## Abstract

In the title compound, C_14_H_7_Cl_2_N_3_O_3_, the dichloro­phenyl and nitro­phenyl rings form dihedral angles of 5.4 (2) and 4.0 (2)°, respectively, with the oxadiazole ring. The nitro group is twisted out of the attached benzene ring by a dihedral angle of 10.4 (3)°. In the crystal, mol­ecules are linked into a chain along the *a* axis by C—H⋯N hydrogen bonds.

## Related literature

For the biological activity of heterocyclic compounds including oxadiazo­les, see: Andersen *et al.* (1994 ▶); Showell *et al.* (1991 ▶); Watjen *et al.* (1989 ▶); Swain *et al.* (1991 ▶); Clitherow *et al.* (1996 ▶); Isloor *et al.* (2010 ▶); Chandrakantha *et al.* (2010 ▶). For related structures, see: Wang *et al.* (2006 ▶); Fun *et al.* (2010*a* ▶,*b* ▶).
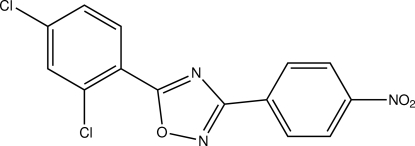


## Experimental

### 

#### Crystal data

C_14_H_7_Cl_2_N_3_O_3_
*M* *_r_* = 336.13
Orthorhombic, 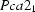

*a* = 13.5272 (5) Å
*b* = 6.5362 (2) Å
*c* = 15.6880 (5) Å
*V* = 1387.08 (8) Å^3^
*Z* = 4
Mo *K*α radiation
μ = 0.48 mm^−1^
*T* = 296 K
0.37 × 0.11 × 0.04 mm

#### Data collection

Bruker SMART APEXII CCD area-detector diffractometer
Absorption correction: multi-scan (*SADABS*; Bruker, 2005 ▶) *T*_min_ = 0.843, *T*_max_ = 0.980
9845 measured reflections
2658 independent reflections
2029 reflections with *I* > 2σ(*I*)
*R*_int_ = 0.045

#### Refinement

*R*[*F* ^2^ > 2σ(*F* ^2^)] = 0.048
*wR*(*F* ^2^) = 0.091
*S* = 1.06
2658 reflections
199 parameters
1 restraint
H-atom parameters constrained
Δρ_max_ = 0.24 e Å^−3^
Δρ_min_ = −0.22 e Å^−3^
Absolute structure: Flack (1983 ▶), 1243 Friedel pairs
Flack parameter: −0.01 (7)

### 

Data collection: *APEX2* (Bruker, 2005 ▶); cell refinement: *SAINT* (Bruker, 2005 ▶); data reduction: *SAINT*; program(s) used to solve structure: *SHELXTL* (Sheldrick, 2008 ▶); program(s) used to refine structure: *SHELXTL*; molecular graphics: *SHELXTL*; software used to prepare material for publication: *SHELXTL* and *PLATON* (Spek, 2009 ▶).

## Supplementary Material

Crystal structure: contains datablocks global, I. DOI: 10.1107/S1600536810011153/ci5063sup1.cif

Structure factors: contains datablocks I. DOI: 10.1107/S1600536810011153/ci5063Isup2.hkl

Additional supplementary materials:  crystallographic information; 3D view; checkCIF report

## Figures and Tables

**Table 1:** Hydrogen-bond geometry (Å, °)

*D*—H⋯*A*	*D*—H	H⋯*A*	*D*⋯*A*	*D*—H⋯*A*
C13—H13*A*⋯N1^i^	0.93	2.54	3.338 (5)	144

Symmetry code: (i) 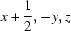.

**Fig. 1.**
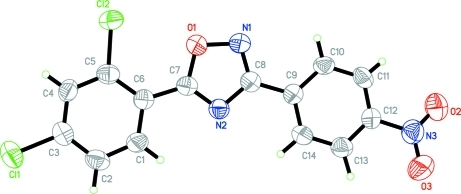
The molecular structure of the title compound, showing 50% probability displacement ellipsoids and the atom-numbering scheme.

**Fig. 2.**
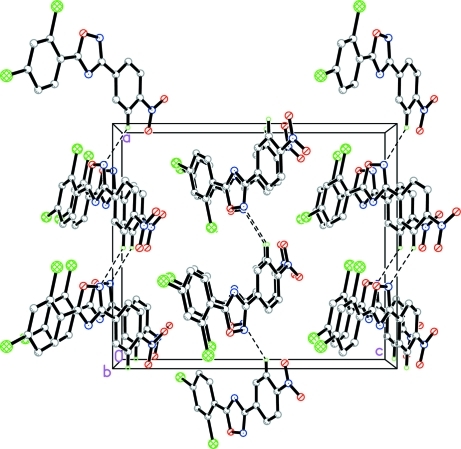
The crystal packing of the title compound, showing hydrogen-bonded (dashed lines) chains along the a axis. H atoms not involved in the interactions have been omitted.

## supplementary crystallographic information

### Comment

Heterocyclic compounds are becoming increasingly important in recent years due to their pharmacological activities (Isloor *et al.*, 2010). Nitrogen- and oxygen-containing five/six-membered heterocyclic compounds are of enormous significance in the field of medicinal chemistry (Chandrakantha *et al.*, 2010). Oxadiazoles play a very vital role in the preparation of various biologically active drugs with anti-inflammatory (Andersen *et al.*,  1994), anti-cancer (Showell *et al.*, 1991), anti-HIV (Watjen *et al.*,  1989), anti-diabetic and anti-microbial (Swain *et al.*,  1991) activities. The results of biological studies showed that oxadiazole derivatives also possess maximum anti-inflammatory, analgesic and minimum ulcerogenic and lipid per-oxidation (Clitherow *et al.*,  1996) properties.

Bond lengths and angles in the title molecule (Fig.1) are within the normal range and comparable to those observed in related structures (Wang *et al.*,  2006; Fun *et al.*, 2010*a*,b). The oxadiazole ring (C7/C8/N1/N2/O1) forms dihedral angles of 5.4 (2)° and 4.0 (2)°, respectively, with with the C1–C6 and C9–C14 benzene rings. The plane of the nitro group is twisted out of the C9–C14 benzene ring by a dihedral of 10.4 (3)°.

In the crystal structure (Fig. 2), the molecules are connected by intermolecular C13—H13A···N1 hydrogen bonds (Table 1) forming chains along the *a* axis.

### Experimental

The title compound was prepared by heating a solution of 2,4-dichlorobenzoyl chloride (1.15 g, 0.02 mol) and N'-hydroxy-4-nitrobenzamidine (1 g, 0.02 mol) in pyridine (30 ml). The reaction mixture was heated at 114°C for 1.5 h and concentrated under vacuum. Further purification was done by column chromatography. The solid obtained was recrystallized using dichloromethane (yield: 1.0 g (55%), m.p 458-461 K).

### Refinement

H atoms were positioned geometrically with C-H = 0.93 Å and were refined using a riding model with U_iso_(H) = 1.2U_eq_(C).

### Crystal data

**Table:**

C_14_H_7_Cl_2_N_3_O_3_	*F*(000) = 680
*M**_r_* = 336.13	*D*_x_ = 1.610 Mg m^−^^3^
Orthorhombic, *P**c**a*2_1_	Mo *K*α radiation, λ = 0.71073 Å
Hall symbol: P 2c -2ac	Cell parameters from 2389 reflections
*a* = 13.5272 (5) Å	θ = 2.6–30.1°
*b* = 6.5362 (2) Å	µ = 0.48 mm^−^^1^
*c* = 15.6880 (5) Å	*T* = 296 K
*V* = 1387.08 (8) Å^3^	Plate, colourless
*Z* = 4	0.37 × 0.11 × 0.04 mm

### Data collection

**Table:**

Bruker SMART APEXII CCD area-detector diffractometer	2658 independent reflections
Radiation source: fine-focus sealed tube	2029 reflections with *I* > 2σ(*I*)
graphite	*R*_int_ = 0.045
φ and ω scans	θ_max_ = 26.0°, θ_min_ = 2.6°
Absorption correction: multi-scan (*SADABS*; Bruker, 2005)	*h* = −13→16
*T*_min_ = 0.843, *T*_max_ = 0.980	*k* = −7→8
9845 measured reflections	*l* = −17→19

### Refinement

**Table:**

Refinement on *F*^2^	Secondary atom site location: difference Fourier map
Least-squares matrix: full	Hydrogen site location: inferred from neighbouring sites
*R*[*F*^2^ > 2σ(*F*^2^)] = 0.048	H-atom parameters constrained
*wR*(*F*^2^) = 0.091	*w* = 1/[σ^2^(*F*_o_^2^) + (0.0361*P*)^2^ + 0.1623*P*] where *P* = (*F*_o_^2^ + 2*F*_c_^2^)/3
*S* = 1.06	(Δ/σ)_max_ = 0.001
2658 reflections	Δρ_max_ = 0.24 e Å^−^^3^
199 parameters	Δρ_min_ = −0.22 e Å^−^^3^
1 restraint	Absolute structure: Flack (1983), 1243 Friedel pairs
Primary atom site location: structure-invariant direct methods	Flack parameter: −0.01 (7)

### Special details

**Table:**

Geometry. All esds (except the esd in the dihedral angle between two l.s. planes) are estimated using the full covariance matrix. The cell esds are taken into account individually in the estimation of esds in distances, angles and torsion angles; correlations between esds in cell parameters are only used when they are defined by crystal symmetry. An approximate (isotropic) treatment of cell esds is used for estimating esds involving l.s. planes.
Refinement. Refinement of F^2^ against ALL reflections. The weighted R-factor wR and goodness of fit S are based on F^2^, conventional R-factors R are based on F, with F set to zero for negative F^2^. The threshold expression of F^2^ > 2sigma(F^2^) is used only for calculating R-factors(gt) etc. and is not relevant to the choice of reflections for refinement. R-factors based on F^2^ are statistically about twice as large as those based on F, and R- factors based on ALL data will be even larger.

### Fractional atomic coordinates and isotropic or equivalent isotropic displacement parameters (Å^2^)

**Table:**

	*x*	*y*	*z*	*U*_iso_*/*U*_eq_	
Cl1	0.37074 (7)	1.17134 (15)	0.20371 (7)	0.0672 (3)	
Cl2	0.08039 (7)	0.73697 (16)	0.34239 (10)	0.0814 (4)	
O1	0.15793 (17)	0.3687 (4)	0.41360 (18)	0.0643 (8)	
O2	0.3673 (2)	−0.6615 (5)	0.6679 (2)	0.0975 (12)	
O3	0.5067 (2)	−0.5915 (5)	0.6109 (2)	0.0959 (11)	
N1	0.1636 (2)	0.1879 (5)	0.4615 (2)	0.0653 (10)	
N2	0.3154 (2)	0.2966 (4)	0.4278 (2)	0.0454 (7)	
N3	0.4200 (3)	−0.5541 (5)	0.6249 (2)	0.0646 (9)	
C1	0.3731 (2)	0.6429 (5)	0.3289 (2)	0.0515 (10)	
H1A	0.4198	0.5472	0.3463	0.062*	
C2	0.4043 (3)	0.8143 (5)	0.2848 (3)	0.0562 (10)	
H2A	0.4709	0.8346	0.2729	0.067*	
C3	0.3342 (3)	0.9546 (5)	0.2589 (2)	0.0478 (9)	
C4	0.2351 (3)	0.9269 (5)	0.2767 (2)	0.0493 (9)	
H4A	0.1888	1.0233	0.2594	0.059*	
C5	0.2060 (2)	0.7551 (5)	0.3204 (2)	0.0484 (9)	
C6	0.2735 (2)	0.6087 (4)	0.3481 (2)	0.0417 (8)	
C7	0.2522 (2)	0.4235 (5)	0.3964 (2)	0.0423 (8)	
C8	0.2581 (3)	0.1536 (5)	0.4676 (2)	0.0434 (8)	
C9	0.2984 (2)	−0.0278 (5)	0.5105 (2)	0.0411 (8)	
C10	0.2372 (2)	−0.1701 (5)	0.5499 (2)	0.0463 (9)	
H10A	0.1692	−0.1493	0.5501	0.056*	
C11	0.2759 (3)	−0.3416 (5)	0.5887 (2)	0.0491 (10)	
H11A	0.2350	−0.4357	0.6158	0.059*	
C12	0.3767 (3)	−0.3693 (5)	0.5861 (2)	0.0457 (9)	
C13	0.4388 (3)	−0.2323 (5)	0.5472 (2)	0.0547 (10)	
H13A	0.5066	−0.2559	0.5458	0.066*	
C14	0.3995 (2)	−0.0590 (5)	0.5103 (2)	0.0508 (9)	
H14A	0.4411	0.0369	0.4851	0.061*	

### Atomic displacement parameters (Å^2^)

**Table:**

	*U*^11^	*U*^22^	*U*^33^	*U*^12^	*U*^13^	*U*^23^
Cl1	0.0778 (7)	0.0595 (6)	0.0642 (6)	−0.0179 (5)	0.0002 (6)	0.0100 (6)
Cl2	0.0403 (4)	0.0675 (6)	0.1363 (11)	−0.0026 (5)	−0.0083 (7)	0.0236 (7)
O1	0.0468 (14)	0.0596 (15)	0.086 (2)	0.0041 (12)	0.0112 (14)	0.0228 (15)
O2	0.089 (2)	0.0758 (19)	0.127 (3)	0.0000 (18)	0.006 (2)	0.045 (2)
O3	0.070 (2)	0.094 (2)	0.124 (3)	0.0193 (18)	−0.001 (2)	0.035 (2)
N1	0.0445 (17)	0.0621 (19)	0.089 (3)	0.0045 (15)	0.0144 (19)	0.0230 (19)
N2	0.0442 (16)	0.0428 (15)	0.0493 (18)	−0.0004 (14)	0.0019 (15)	−0.0026 (15)
N3	0.066 (2)	0.061 (2)	0.066 (2)	0.0007 (19)	−0.007 (2)	0.0065 (19)
C1	0.046 (2)	0.0437 (19)	0.065 (3)	0.0046 (14)	0.0124 (19)	−0.0034 (18)
C2	0.051 (2)	0.054 (2)	0.065 (3)	−0.0046 (18)	0.015 (2)	−0.009 (2)
C3	0.061 (2)	0.044 (2)	0.038 (2)	−0.0077 (17)	0.0005 (18)	−0.0042 (17)
C4	0.050 (2)	0.0450 (19)	0.053 (2)	0.0004 (16)	−0.0104 (18)	0.0052 (18)
C5	0.0418 (18)	0.0520 (19)	0.052 (2)	−0.0054 (16)	−0.0026 (17)	−0.0072 (19)
C6	0.0440 (18)	0.0357 (15)	0.046 (2)	0.0000 (13)	0.0014 (17)	−0.0082 (18)
C7	0.0402 (18)	0.0428 (17)	0.044 (2)	−0.0013 (16)	0.0029 (17)	−0.0085 (17)
C8	0.0472 (19)	0.0424 (17)	0.040 (2)	−0.0011 (17)	0.0037 (19)	−0.0072 (16)
C9	0.0462 (19)	0.0400 (17)	0.0370 (19)	0.0007 (15)	0.0012 (17)	−0.0081 (16)
C10	0.0367 (18)	0.052 (2)	0.050 (2)	0.0023 (16)	0.0099 (17)	−0.0048 (18)
C11	0.054 (2)	0.0464 (19)	0.047 (2)	−0.0071 (16)	0.0106 (19)	0.0000 (18)
C12	0.050 (2)	0.0432 (19)	0.043 (2)	−0.0008 (16)	−0.0008 (17)	0.0002 (17)
C13	0.045 (2)	0.059 (2)	0.060 (3)	−0.0018 (18)	−0.0032 (19)	0.006 (2)
C14	0.044 (2)	0.053 (2)	0.055 (2)	−0.0082 (16)	0.0048 (18)	0.0032 (19)

### Geometric parameters (Å, °)

**Table:**

Cl1—C3	1.732 (3)	C4—C5	1.373 (5)
Cl2—C5	1.739 (3)	C4—H4A	0.93
O1—C7	1.352 (4)	C5—C6	1.392 (4)
O1—N1	1.403 (4)	C6—C7	1.458 (4)
O2—N3	1.207 (4)	C8—C9	1.468 (4)
O3—N3	1.218 (4)	C9—C14	1.384 (4)
N1—C8	1.302 (5)	C9—C10	1.390 (4)
N2—C7	1.289 (4)	C10—C11	1.378 (4)
N2—C8	1.366 (4)	C10—H10A	0.93
N3—C12	1.474 (5)	C11—C12	1.377 (5)
C1—C2	1.383 (5)	C11—H11A	0.93
C1—C6	1.398 (4)	C12—C13	1.371 (5)
C1—H1A	0.93	C13—C14	1.379 (5)
C2—C3	1.380 (5)	C13—H13A	0.93
C2—H2A	0.93	C14—H14A	0.93
C3—C4	1.381 (5)		
			
C7—O1—N1	106.2 (2)	N2—C7—O1	112.3 (3)
C8—N1—O1	103.8 (3)	N2—C7—C6	127.0 (3)
C7—N2—C8	103.8 (3)	O1—C7—C6	120.7 (3)
O2—N3—O3	123.6 (4)	N1—C8—N2	113.9 (3)
O2—N3—C12	118.2 (3)	N1—C8—C9	122.5 (3)
O3—N3—C12	118.2 (4)	N2—C8—C9	123.5 (3)
C2—C1—C6	122.1 (3)	C14—C9—C10	119.4 (3)
C2—C1—H1A	119.0	C14—C9—C8	119.0 (3)
C6—C1—H1A	119.0	C10—C9—C8	121.6 (3)
C3—C2—C1	118.4 (3)	C11—C10—C9	121.0 (3)
C3—C2—H2A	120.8	C11—C10—H10A	119.5
C1—C2—H2A	120.8	C9—C10—H10A	119.5
C2—C3—C4	121.3 (3)	C10—C11—C12	118.0 (3)
C2—C3—Cl1	119.6 (3)	C10—C11—H11A	121.0
C4—C3—Cl1	119.1 (3)	C12—C11—H11A	121.0
C5—C4—C3	119.1 (3)	C13—C12—C11	122.3 (3)
C5—C4—H4A	120.4	C13—C12—N3	118.4 (3)
C3—C4—H4A	120.4	C11—C12—N3	119.3 (3)
C4—C5—C6	122.0 (3)	C12—C13—C14	119.2 (3)
C4—C5—Cl2	115.8 (3)	C12—C13—H13A	120.4
C6—C5—Cl2	122.1 (3)	C14—C13—H13A	120.4
C5—C6—C1	117.0 (3)	C13—C14—C9	120.1 (3)
C5—C6—C7	127.1 (3)	C13—C14—H14A	120.0
C1—C6—C7	115.8 (3)	C9—C14—H14A	120.0
			
C7—O1—N1—C8	−0.2 (4)	O1—N1—C8—N2	0.1 (4)
C6—C1—C2—C3	−0.3 (5)	O1—N1—C8—C9	−177.5 (3)
C1—C2—C3—C4	0.4 (5)	C7—N2—C8—N1	0.2 (4)
C1—C2—C3—Cl1	179.9 (3)	C7—N2—C8—C9	177.7 (3)
C2—C3—C4—C5	−0.6 (5)	N1—C8—C9—C14	176.5 (4)
Cl1—C3—C4—C5	179.9 (3)	N2—C8—C9—C14	−0.8 (5)
C3—C4—C5—C6	0.7 (5)	N1—C8—C9—C10	−2.4 (5)
C3—C4—C5—Cl2	178.3 (3)	N2—C8—C9—C10	−179.7 (3)
C4—C5—C6—C1	−0.6 (5)	C14—C9—C10—C11	0.0 (5)
Cl2—C5—C6—C1	−178.0 (3)	C8—C9—C10—C11	178.9 (3)
C4—C5—C6—C7	178.5 (3)	C9—C10—C11—C12	−1.0 (5)
Cl2—C5—C6—C7	1.0 (5)	C10—C11—C12—C13	0.6 (5)
C2—C1—C6—C5	0.4 (5)	C10—C11—C12—N3	−178.3 (3)
C2—C1—C6—C7	−178.8 (3)	O2—N3—C12—C13	171.1 (4)
C8—N2—C7—O1	−0.3 (4)	O3—N3—C12—C13	−9.9 (6)
C8—N2—C7—C6	179.1 (3)	O2—N3—C12—C11	−9.9 (5)
N1—O1—C7—N2	0.4 (4)	O3—N3—C12—C11	169.0 (4)
N1—O1—C7—C6	−179.1 (3)	C11—C12—C13—C14	0.8 (6)
C5—C6—C7—N2	−174.0 (4)	N3—C12—C13—C14	179.7 (3)
C1—C6—C7—N2	5.0 (5)	C12—C13—C14—C9	−1.8 (6)
C5—C6—C7—O1	5.3 (5)	C10—C9—C14—C13	1.4 (6)
C1—C6—C7—O1	−175.6 (3)	C8—C9—C14—C13	−177.5 (3)

### Hydrogen-bond geometry (Å, °)

**Table:**

*D*—H···*A*	*D*—H	H···*A*	*D*···*A*	*D*—H···*A*
C13—H13A···N1^i^	0.93	2.54	3.338 (5)	144

Symmetry codes: (i) *x*+1/2, −*y*, *z*.
